# The presence of serum anti-*Ascaris lumbricoides *IgE antibodies and of *Trichuris trichiura *infection are risk factors for wheezing and/or atopy in preschool-aged Brazilian children

**DOI:** 10.1186/1465-9921-11-114

**Published:** 2010-08-23

**Authors:** Neuza M Alcântara-Neves, Samuel J Badaró, Mariese CA dos Santos, Lain Pontes-de-Carvalho, Maurício L Barreto

**Affiliations:** 1 Departmento de Ciências da Biointeração, Instituto de Ciências da Saúde, Universidade Federal da Bahia, Salvador, Brazil; 2 Laboratório de Patologia e Biologia Interativa, Centro de Pesquisas Gonçalo Moniz, Fundação Oswaldo Cruz, Salvador, Brazil; 3 Instituto de Saúde Coletiva, Universidade Federal da Bahia, Salvador, Brazil

## Abstract

**Background:**

The elucidation of factors that trigger the development of transient wheezing in early childhood may be an important step toward understanding the pathogenesis of asthma and other allergic diseases later in life. Transient wheezing has been mainly attributed to viral infections, although sensitisation to aeroallergens and food allergens may occur at an early age. In developing countries, intestinal helminthic infections have also been associated with allergy or atopy-related disorders.

**Objective:**

The aim of this study was to explore the association of *Trichuris trichiura *and *Ascaris lumbricoides *infections with wheezing and atopy in early childhood.

**Study design:**

A cross-sectional study using a Portuguese-language ISAAC phase I questionnaire, adapted for preschool-aged children, nested in a cohort study of childhood diarrhoea, was conducted on 682 children. Two faecal samples per child were examined for the presence of intestinal helminthic infection. IgE antibodies against three allergenic preparations *(Dermatophagoides pteronyssinus, Blomia tropicalis *and common child food), as well as against *A. lumbricoides *antigens, were measured in a sub-sample of these children, whose parents allowed the procedure. Atopy was defined by the presence of levels of serum IgE antibodies ≥0.35 kU/L against at least one of the three tested allergenic preparations.

**Results:**

Active *T. trichiura *infection but not *A. lumbricoides *infection was positively associated with wheezing in the total studied children population [adjusted OR = 2.60; CI = 1.54;4.38] and in the atopic children sub-population [adjusted OR = 3.07; CI = 1.00;9.43]. The association with atopy was also positive and statistically significant only in the brute analysis [OR = 2.13; CI = 1.03;4.40]. Anti-*A. lumbricoides *IgE antibodies, but not current *A. lumbricoides *infection, were positively associated with wheezing in atopic children [adjusted OR = 2.01; CI = 1.00;4.50] and in non-atopic children [adjusted OR = 3.07; CI = 1.13;8.35] and it was also associated with atopy [adjusted OR = 7.29; CI = 3.90; 13.4]. On the other hands, reports of wheezing were not significantly associated with atopy.

**Conclusions:**

These data corroborate previous studies showing that wheezing is predominantly associated with infection in early childhood and shows that anti-*A. lumbricoides *IgE antibodies, but not active *Ascaris *infections, are associated with wheezing and atopy. Additionally, the data demonstrate that *T. trichiura *infection may play a role in the pathogenesis of atopic wheezing in early childhood.

## Introduction

The pathogenesis of allergy is multifactorial, depending on both the individual's genetic background and environmental factors. The prevalence and incidence of allergic diseases are increasing worldwide, particularly in the urban populations of developed countries. The International Study of Asthma and Allergies in Childhood (ISAAC), conducted in several Brazilian cities, showed that the city of Salvador, where the present work was completed, had a very high prevalence (24.0%) of wheezing among children [1]. Among the environmental factors that may trigger allergic diseases, infections are the most studied. Surprisingly, helminthic infections, which induce the host immune system to mount T helper 2 (Th2) immune responses, as during an allergic reaction, have been negatively associated with atopy and allergic illness [2,3]. In spite of the evidence that helminths protect their hosts against allergy, some studies have indicated that infection by these parasites does not affect allergy or atopy [4,5], while others have demonstrated a positive association of these infections with atopy as well as with asthma and rhinitis [6,7]. Worm burden and the timing of infection seem to play a role in the relationship between parasite infection and the host immune response [8]. Recently, our research group demonstrated that infection by *Trichuris trichiura *during early childhood was negatively associated with the development of aeroallergen skin test reactivity in later childhood [9]. In the present work, we report that *Ascaris lumbricoides *sensitisation and *T. trichiura *infection, but not *A. lumbricoides *infection, were associated with wheezing in early childhood. In addition, *A. lumbricoides *sensitisation was also strongly associated with atopy.

## Materials and methods

### Study population

The study population was part of a cohort of 1283 children, born from 1998 to 2000, and recruited between 2000-2001, living in 24 small geographical areas (also called sentinel areas) in Salvador, a major city in the northeast of Brazil. These children were originally selected for a study to evaluate the impact of a sanitation program on the incidence of childhood diarrhoea [10,11] and were followed up to 2002. The populations of these areas are of low socioeconomic status and similar ethnicity. The present data were collected from a randomly selected sub-sample of 683 children enrolled in the cohort, all of them were less than five years old (average age of 2.3 years, range from 1 - 4.2 years). This sub-sample size was estimated assuming a 22% prevalence for wheezing and a 25% prevalence for infection by intestinal helminthes, based on on-going studies carried out on children of the same city, with 3% precision and 95% confidence. Demographic and social data were collected using a validated questionnaire. The children were visited twice per week for one year to collect information on the occurrence of diarrhoea. At the end of the study, a Portuguese-language ISAAC phase I questionnaire, adapted for young children, was administered to the children's parents or guardians by trained interviewers. Two stool samples were collected two days apart and examined for parasitic infections. Blood was collected from a subgroup of 283 children whose parents agreed to the procedure. Serum aliquots prepared from the blood samples were stored at -70°C until use. The sera were then assessed for IgE antibodies against house dust mites, common childhood food allergens (milk, ovalbumin and peanut) and *A. lumbricoides *antigens. All parasite-infected children received anti-helminthic and anti-protozoan treatment and were sent for ambulatory treatment during episodes of asthma and diarrhoea. Ethical approval was granted by the Ethical Committee on Research of the Instituto de Saúde Coletiva, Universidade Federal da Bahia, and written informed consent was obtained from the children's parents or guardians.

### Wheezing and atopy definitions

Wheezing was defined as four or more episodes of wheeze associated with cough, shortness of breath so intense as to interrupt sleep, each lasting at least three days, and treatment for asthma medication by a doctor in the past 12 months.

Atopy was defined by the presence of serum IgE antibodies ≥0.35 kU/L against at least one of the following allergenic preparations: *Dermatophagoides pteronyssinus*, *Blomia tropicalis*, and a mixture of allergenic foods commonly consumed by children (milk, ovalbumin and peanuts).

### Identification and quantification of helminthic eggs in stool samples and diarrhoea

Faecal samples were analysed by a sedimentation method [12] and by the Kato-Katz's thick-smear technique [13] for detecting and counting helminths (*T. trichiura*, *A. lumbricoides*, hookworm, and *Schistosoma mansoni*) eggs. Because only a few children were infected with *S. mansoni *and hookworms, these parasites were not considered in the analysis. Children were deemed infected when at least one faecal sample tested was positive for the helminths of interest. The number of days of diarrhoea was obtained in the follow-up period.

### IgE antibody quantification

IgE antibodies against common childhood foods, *D. pteronyssinus*, *B. tropicalis*, and *A. lumbricoides *were measured using the Immunocap System IgE FEIA (Pharmacia, Upsala, Sweden) according to the manufacturer's instructions. Serum samples containing ≥0.35 kU/L or more of IgE antibodies were considered positive. These allergens were chosen based on previous observations, in paediatric clinics in Salvador, that they are the most common sensitising agents in young children in this city. These mites are also the most common sensitising agents in older children in this city [9].

### Statistical analysis

The work design was cross-sectional, for studying wheezing and atopy (outcomes) in early childhood, nested in a cohort study of childhood diarrhoea. The variables analysed for their association with the outcomes were: sex; age; parental asthma; maternal literacy; presence of a septic system in the house; infection with *A. lumbricoides or T. trichiura *(as indicated not only by the presence of eggs in the stool but also by their numbers) and presence of anti-*A. lumbricoides IgE *antibodies. Occurrence of diarrhoea was presented as incidence data. Logistic univariate analysis was performed to compare each variable with the studied outcomes (wheezing and specific IgE) by calculating odds ratios [ORs and 95% confidence interval]. To choose whether it would be included in the final logistic regression model, a variable that was not statistically significant in the univariate analysis was tested separately. If this variable continued to be not significant, it was removed from the model, while if it became significant, it was retained. All variables were retested until only significant risk factors and *a priori *variables remained. Because a sub-sample was taken from the 682 studied children to be tested for specific IgE antibodies, the Pearson Chi^2 ^test was used to analyze possible differences between the sub-group of the study population that was tested for IgE antibodies (283 children) and the sub-group that was not tested (399 children). Since the children were clustered in sentinel areas, the data were adjusted by the sentinel areas in the preliminary analysis, using multivariate logistic analyses, to avoid a clustering effect. However, as no difference among sentinel areas was observed, this variable was dropped from the final analysis. The associations between all variables and wheezing or atopy were finally investigated using a multivariate logistic regression model that adjusted for the variables mentioned above. The Spearman's linear correlation coefficient between *A. lumbricoides *eggs in stool and the serum concentration of anti-*A. lumbricoides *IgE antibodies was calculated.

## Results

The ISAAC phase I questionnaire was administered to 682 children and specific IgE measurements were performed on the sera of 283 of these children. Comparisons among those tested for IgE antibodies and among those in the untested group showed that the frequencies of all studied variables were similar between the two groups (p > 0.05), except for the incidence of diarrhoea ≥6 days, which was slightly lower in the tested group than in the untested group (35.1% and 39.5% respectively; p = 0.02; data not shown).

Table 1 shows the results of univariate and multivariate analyses of associations between wheezing and several variables (sex, age, maternal literacy, presence of a septic system, *A. lumbricoides *infection, *T. trichiura *infection, and incidence of diarrhoea) in the 682 studied children. No associations were identified between wheezing and age or *A. lumbricoides *infection. Males demonstrated the highest frequency of wheezing over the past 12 months, as shown by multivariate analysis [adjusted OR = 1.55; CI = 1.12;2.14]. On the other hand, maternal literacy was found to be negatively associated with wheezing by both univariate and multivariate analyses, but this association was statistically significant only in the univariate analysis [OR = 0.61; CI = 0.40;0.92]. The lack of a household connection to the sewer system was associated with high frequency of wheezing, as shown by univariate and multivariate analyses [OR = 1.47; CI = 1.08;2.01; adjusted OR = 1.43; CI = 1.04;1.97]. *T. trichiura *infection was also positively associated with wheezing by both analyses [OR = 2.99; CI = 1.85;4.84; adjusted OR = 2.60; CI = 1.54;4.38], while the number of days of diarrhoea greater than six was positively associated with wheezing by both analyses, but this association was statistically significant only by univariate analysis [OR = 1.58; CI = 1.04;2.40; adjusted OR = 1.40; CI = 0.89-2.21].

**Table 1:** Univariate and multivariate analyses of the association of reported wheezing over the past 12 months with gender, age, intestinal helminthic infection, and diarrhoea in the studied population

	Wheezing				
	
Variables	N = 682	Yes (257)	No (425)	OR	Adjusted OR
	n (%)	n (%)	N (%)	[95% CI]	[95% CI]
**Gender**					
Female	317 (46.5)	100 (31.5)	217 (68.5)	1^#^	1
Male	365 (53.5)	157 (43.0)	208 (57.0)	1.64 [1.20;2.24]	1.55 [1.12;2.14]
**Age (months)**					
19 to 50	326 (47.8)	120 (36.8)	206 (63.2)	1	1
0 to 18	356 (52.2)	137 (38.5)	219 (61.5)	1.07 [0.79;1.46]	1.07 [0.76;1.51]
**Maternal literacy**					
Illiterate/primary	159 (23.3)	71 (44.7)	88 (55.3)	1	1
Secondary (incomplete)	278 (40.8)	106 (38.1)	172 (61.9)	0.76 [0.51;1.13]	0.79 [0.53;1.20]
Secondary (complete)	245 (35.9)	80 (32.7)	165 (67.3)	0.61 [0.40;0.92]	0.72 [0.47;1.11]
**Sewer system**					
Yes	341 (50.0)	113 (33.1)	228 (66.9)	1	1
No	341 (50.0)	144 (42.2)	197 (57.8)	1.47 [108;2.01]	1.43 [1.04;1.97]
***T. trichiura *eggs**					
No	602 (88.3)	208 (34.6)	394 (65.4)	1	1
Yes	80 (11.7)	49 (61.2)	31 (38.8)	**2.99 [1.85;4.84]**	**2.60 [1.54;4.38]**
***A. lumbricoides *eggs**					
No	544 (79.8)	196 (36.0)	348 (64.0)	1	1
Yes	138 (20.2)	61 (44.2)	77 (55.8)	1.41 [0.93;2.05]	1.09 [0.71;1.66]
**Days with diarrhoea**					
None	157 (23.0)	50 (31.8)	107 (68.2)	1	1
<6 days	273 (40.0)	100 (36.6)	173 (63.4)	1.24 [0.82;1.88]	1.21[0.79;1.87]
≥6 days	252 (37.0)	107 (42.5)	145 (57.5)	1.58 [1.04;2.40]	1.40 [0.89;2.21]

^# ^Class reference of the variable. Bold numbers are statistically significant at 0.05.

Table 2 demonstrates that of the 283 children who were examined for IgE antibodies against aeroallergens and food allergens, 48.2% had circulating IgE antibodies ≥0.35 kU/L and 10.0% had levels ≥3.5 kU/L against at least one of these allergens. The most frequent anti-allergen IgE antibody detected was anti-*B. tropicalis *(36.1%), followed by anti-food (23.9%) and anti-*D. pteronyssinus (*19.3%). Anti-*A. lumbricoides *IgE antibodies occurred in 35% of the samples and their levels did not correlate with the number of *A. lumbricoides *eggs found in the stool (Spearman's linear correlation coefficient: r = 0.05; p = 0.255). In fact, 22 out of 53 (41.5%) children in whose stools *A. lumbricoides *eggs were detected, had no serum IgE antibodies against this worm antigens.

**Table 2:** Frequencies of anti-allergen and anti-*A. lumbricoides *serum IgE antibodies in the studied 283 pre-school-aged children

IgE antibodies against	Antibody concentration	
	
	≥0.35 kU/L	≥3.5 kU/L
	
	n (%)	n (%)
Common childhood foods	67 (23.9)	7 (2.5)
*D. pteronyssinus*	54 (19.3)	8 (2.9)
*B. tropicalis *	101 (36.1)	20 (7.1)
Common childhood foods or *B. tropicalis *or *D. pteronyssinus*	135 (48.2)	28 (10.0)
*A. lumbricoides*	99 (35.4)	25 (8.9)

In the subgroup of children which was positive for circulating anti-allergen IgE antibodies, positive associations were found between wheezing over the past 12 months and *T. trichuris *infection in both brute [OR = 2.69; CI = 1.07;6.77] and adjusted analyses [OR = 3.07; CI = 1.00;9.43]. Anti-*A. lumbricoides *IgE antibody levels were positively associated with wheezing in these atopic children, with borderline non-significance in the univariated [OR =1.89; CI = 0.93;3.84] and significantly associated in the multivariate analyses [adjusted OR =2.01; CI = 1.00;4.50] (Table 3). Wheezing in non-atopic children was positive but not significantly associated with *T. trichiura *infection in univariate and multivariate analyses [OR = 2.77; CI = 0.88;8.76; adjusted OR = 2.45; CI = 0.70;8.53] and not associated with *A. lumbricoides *infection [OR = 1.17; CI = 0.48;2.86; adjusted OR = 0.76; CI = 0.27;2.11], but was positive and statistically associated with anti-*A. lumbricoides *IgE [OR = 2.80; CI = 1.13;6.94; adjusted OR = 3.07; CI = 1.13;8.35] (Table 4).

**Table 3:** Univariate and multivariate analyses of the association of wheezing over the past 12 months with intestinal helminthic infections, anti-*A. lumbricoides *IgE antibodies and diarrhoea in 135 atopic children

Wheezing in atopics*					
	
Variables	N = 135 n (%)	Yes (55) n (%)	No (80) n (%)	OR [95%CI]	Adjusted** OR [95%CI]
***T. trichuris***					
No	112 (83.0)	41 (36.6)	71 (63.4)	1^#^	1
Yes	23 (17.0)	14 (60.9)	9 (39.1)	**2.69[1.07;6.77]**	**3.07[1.00;9.43]**
***Ascaris***					
No	108 (80.0)	44 (40.7)	64 (59.3)	1	1
Yes	27 (20.0%)	11 (40.7)	16 (59.3)	1.00[0.42;2.36]	0.44[0.15;1.32]
**Anti-*Ascaris *IgE**					
<0.35 kU/L	59 (43,7)	19 (32,2)	40 (67,8)		
≥0.35 kU/L	76 (56,3)	36 (47,4)	40 (52,6)	1,89[0,93;3,84]	**2.01[1.00;4.50]**
**Diarrhoea**					
None	27 (20.0)	9 (33.3)	18 (66.7)	1	1
<6 days	59 (43.7)	21 (35.6)	38 (64.4)	1.10[0.42;2.89	0.97[0.35;2,70]
≥6 days	49 (36.3)	25 (51.0)	24 (49.0)	2.08[0.78;5.53]	1.62[0.53;4.89]

*Atopy defined as the presence of serum IgE antibodies against at least one of the following allergens: *D. pteronyssinus*, *B. tropicalis*, mlk, ovalbumin, and peanuts.

**Adjusted for gender, age, *T. trichiura *eggs, *A. lumbricoides *eggs, days with diarrhoea and anti-*Ascaris *IgE.

^# ^Class reference of the variable.

Bold numbers are statistically significant at 0.05.

**Table 4:** Univariate and multivariate analyses of the association of wheezing over the past 12 months with intestinal helminthic infections, anti-*A. lumbricoides *IgE antibodies and diarrhoea in 148 non-atopic children

Variables	Wheezing in non-atopics*				
	
	N = 148	Yes (47)	No (101)	OR	Adjusted** OR
	n(%)	n (%)	n (%)	[95%CI]	[95%CI]
***T. trichuris***					
No	135 (91.2)	40 (29.6)	95 (70.4)	1^#^	1
Yes	13 (8.8)	7 (53.8)	6 (46.2)	2.77[0.88;8.76]	2.45[0.70;8.53]
***Ascaris***					
No	122 (82.4)	38 (31.1)	84 (68.9)	1	1
Yes	26 (17.6)	9 (34.6)	17 (65.4)	1.17[0.48;2.86]	0.76[0.27;2.11]
**Anti-*Ascaris *IgE**					
<0.35 kU/L	125 (84.5)	35 (28.0)	90 (72.0)		
≥0.35 kU/L	23 (15.5)	12 (52.2)	11 (47.8)	2.80[1.13;6.94]	3.07[1.13;8.35]
**Diarrhoea**					
None	23 (15.5)	7 (30.4)	16 (69.6)	1	1
<6 days	62 (41.9)	18 (29.0)	44 (71.0)	0.93[0.33;2.66]	1.17[0.39;3.53]
≥6 days	63 (42.6)	22 (34.9)	41 (65.1)	1.23[0.44;3.43]	1.79[0.55;5.78]

*Atopy defined as the presence of serum IgE antibodies against at least one of the following allergens: *D. pteronyssinus*, *B. tropicalis*, mlk, ovalbumin, and peanuts.

**Adjusted for gender, age, *T. trichiura *eggs, *A. lumbricoides *eggs and days with diarrhoea.

^# ^Class reference of the variable.

Bold numbers are statistically significant at 0.05.

The associations between the studied variables and atopy (defined as the presence of serum IgE antibodies ≥0.35 kU/L against at least one of the studied allergens) are shown in Table 5. The presence of *A. lumbricoides *eggs in the stool was not associated with atopy by either the univariate or the multivariate analysis [OR = 1.17; CI = 0.65;2.13; adjusted OR = 0.64; CI = 0.30;1.39], while there was an association of anti-*A. lumbricoides *IgE antibodies with this outcome by both types of analyses [OR = 7.00; CI = 4.00;12.25; adjusted OR = 7.29; CI = 3.90;13.64]. The presence of *T. trichiura *eggs in the stool was also positively associated with atopy in both analyses but it was statistically significant only in the univariate analysis [OR = 2.13; CI = 1.03;4.40; adjusted OR = 1.52; CI = 0.66-3.50]. Reported wheezing over the previous 12 months and the occurrence of diarrhoea was not associated with atopy by either univariate or multivariate analyses. The A. *lumbricoides *and *T. trichiura *egg burdens of these children were low to moderate, according to WHO cut-offs [14], and no statistical associations were found between this variable and wheezing or atopy (data not shown).

**Table 5:** Univariate and multivariate analyses of the association of atopy with gender, age, intestinal helminthic infections, and diarrhoea in the subpopulation tested for serum anti-allergen IgE antibodies

Variables		Atopy*			
		
	N = 283	Yes (135)	No (148)	OR	^**##**^**Adjusted OR**
	n (%)	n (%)	n (%)	[95% CI]	[95%CI]
**Gender**					
Female	129 (45.6)	57 (44.2)	72 (55.8)	1^#^	1
Male	154 (54.4)	78 (50.6)	76 (49.4)	1.30 [0.81;2.07]	1.30 [0.76;2.24]
**Age (months)**					
19 a 48	137 (48.4)	76 (55.5)	61 (44.5)	1	1
0 a 18	146 (51.6)	59 (40.4)	87 (59.6)	0.54 [0.34;0.87]	0.64 [0.36;1.15]
**Maternal literacy**					
Illiterate/primary	60 (21.2)	32 (53.3)	28 (46.7)	1	1
Secondary incomplete	129 (45.6)	57 (44.2)	72 (55.8)	0.69 [0.37;1.28]	0.84 [0.41;1. 70]
Secondary complete or more	94 (33.2)	46 (48.9)	48 (51.1)	0.84 [0.44;1.60]	1.16 [0.55;2.45]
**Sewer system**					
Yes	141 (49.8)	62 (44.0)	79(56.0)	1	1
No	142 (50.2)	73 (51.4)	69 (48.6)	1.35 [0.84;2.15]	1.40 [0.82;2.37]
***Trichuris e*ggs**					
No	247 (87.3)	112 (45.3)	135 (54.7)	1	1
Yes	36 (12.7)	23 (63.9)	13 (36.1)	2.13 [1.03;4.40]	1.52 [0.66;3.50]
***Ascaris *eggs**					
No	230 (81.3)	108 (47.0)	122 (53.0)	1	1
Yes	53 (18.7)	27 (50.9)	26 (49.1)	1.17 [0.65;2.13]	0.64 [0.30;1.39]
**Anti-*Ascaris *IgE**					
<0.35 KU/L	184 (65,0)	59 (32,1)	125 (67,9)	1	1
≥0.35 KU/L	99 (35,0)	76 (76,8)	23 (23,2)	7.00 [4.00;12.25]	7.29 [3.90;13.64]
**Wheezing in the** **past 12 months**					
No	181 (64.0)	80 (44.2)	101 (55.8)	1	1
Yes	102 (36.0)	55 (53.9)	47 (46.1)	1.48 [0.91;2.41]	1.02 [0.57;1.80]
**Days with diarrhoea**					
None	50 (17.7)	27 (54.0)	23 (46.0)	1	1
<6 days of duration	121 (42.8)	59 (48.8)	62 (51.2)	0.81 [0.42;1.57]	0.84 [0.40;1.80]
≥6 days of duration	112 (39.6)	49 (43.8)	63 (56.2)	0.66 [0.34; 1.29]	0.99 [0.44;2.24]

*Defined as the presence of serum IgE antibodies against at least one of the following allergens: *D. pteronyssinus*, *B. tropicalis*, mlk, ovalbumin, and peanuts.

**Adjusted for gender, age, *T. trichiura *eggs, *A. lumbricoides *eggs and days with diarrhoea.

^#^Class reference of the variable.

Bold numbers are statistically significant at 0.05.

## Discussion

In the present study, although 39% of the 283 studied children for IgE antibodies experienced at least one episode of severe wheezing over the past 12 months, only 10% possessed IgE antibodies against common allergens ≥3.5 kU/L. This IgE level is considered as the threshold for developing the clinical symptoms of allergic asthma [15]. Furthermore, no association was found of wheezing with anti-allergen IgE antibody levels ≥0.35 kU/L (Table 5). Thus, most of the reported wheezing episodes were not atopy-related. Further support for this observation comes from the finding that the absence of a household connection to the sewer system increased the odds of wheezing in the multivariate analyses. This finding seems contrary to the hygiene hypothesis but reinforces the possibility of an infectious aetiology for the reported wheezing. The current study is in agreement with previous reports that most early-childhood wheezing is not related to allergy, based on data from developed countries, where this condition is mainly described associated with virus infection [16,17]. The finding that wheezing is more common in boys than in girls is also in accordance with previous studies [16].

Both *A. lumbricoides *and *T. trichiura *infections were positively associated with wheezing in the present study (in the case of *A. lumbricoides*, only when the infection was serologically defined by the presence of specific IgE antibodies). The associations were robust, even after adjustment for confounding variables in the multiple logistic regression analysis. The finding that anti-*A. lumbricoides *IgE antibodies increases the risk for atopy and wheezing has been reported previously in regions of low, medium, and high endemicity [18-20]. On the other hand, *T*. *trichiura *infection has not been described as positively associated with asthma or atopy in the literature, although an association of this parasite with atopic eczema has been reported [21]. Intestinal helminths induce IgE-producing, Th2 immune responses [22]. The *A. lumbricoides *infection that leads to high levels of anti-*Ascaris *antibodies could also promote, by a non-specific effect, higher levels of allergen-specific antibodies [23,24]. This could explain the association between this parasite specific IgE antibodies and atopic wheezing, but not with non-atopic wheezing. An explanation for this last association could be the sensitization of lung mast cells with anti-*A. lumbricoides *IgE antibodies and their eventual degranulation by antigens released by migrating *A. lumbricoides *larvae.

The presence of high levels of anti-*A. lumbricoides *IgE antibodies would reflect at least a partial immunity to infection, which would explain the association of wheezing with IgE antibodies and not with *A. lumbricoides *eggs in the stool. This immunity would therefore explain the absence of correlation between *A. lumbricoides *eggs in the stool and this parasite specific serum IgE antibodies, observed in this and in a previously reported study [25]. The frequency of antibody-eliciting larvae invasion should be directly proportional to poor sanitation, such as a lack of a sewage system, which was also associated with more frequent wheezing in the studied population.

In addition to association with wheezing, we identified a clear association between the presence of anti-*A. lumbricoides *IgE antibodies and atopy. However, as mentioned earlier, there are conflicting reports on intestinal helminthic infections decreasing, not affecting or increasing atopy and allergic symptoms [2-7], although a systematic review and meta-analysis found that *A. lumbricoides *infection was associated with a significantly increased risk of asthma [26]. The fact that *A. lumbricoides *eggs were not associated with wheezing and atopy, while there were strong associations of anti-*A. lumbricoides *IgE antibodies with these two outcomes, may explain why some previous studies relying only on the detection of helminthic eggs did not identify these associations [4,5]. In addition, one widely accepted hypothesis that has been put forward to explain these discrepant findings is that high worm burden and chronic helminthic infections would lead to the production of immunoregulatory cytokines and to protection against atopy and asthma, while low worm burden or recent infection would promote atopy and allergy [5,8]. The present data corroborate this hypothesis, since the studied children are very young and probably had been infected recently. Moreover, these children had low to moderate *A. lumbricoides *and *T. trichiura *egg burdens. It is possible that several months or years in the future, with repeated or chronic *T. trichiura *infections, the tendency would be for infection-associated regulatory cells [27,28] reducing atopy. This phenomenon would account for the findings of an investigation carried out recently in Salvador by our research group [9], which showed that early infection by *T. trichiura *protected against the development of aeroallergen skin test reactivity in later childhood. This finding in older children, and the demonstration in the present report of a positive association between *T. trichiura *infection and wheezing in infants, is consistent with the acute-chronic infection hypothesis explaining different types of association between helminth infection and atopy, mentioned above.

In the present work, we have quantified parasitic eggs in faecal samples and analysed the association of worm burden with wheezing and atopy (data not shown). The absence of association between worm burden with wheezing and atopy was attributed to a low variability of parasite burdens, since all children had low to moderate egg intensity.

Unfortunatelly, anti-*T. trichiura *IgE antibodies could not be measured in this work, so it was not possible to study the association of IgE antibodies against this parasite with the studied outcomes, as was done for anti-*A. lumbricoides *IgE levels. The hypothesis proposed above, however, predicts that the association of wheezing and atopy with anti-*T. trichiura *IgE antibodies should be stronger than their association with *T. trichiura *egg burden. The presence of a statistically significant association of *T. trichiura *infection with atopic wheezing and not with atopy, in the multivariate analysis, is not easily explained. However, it may indicate that *T. trichiura *infection would enhance the development of wheezing more easily in children who are more susceptible to develop lung inflammation, perhaps by stimulating on-going allergic responses. In addition, another intriguing question remains. Since anti-*A. lumbricoides *IgE antibodies, but not *A. lumbricoides *eggs, are associated with wheezing and atopy in the studied children, why is the presence of *T. trichiura *eggs in the stool robustly associated with wheezing in the same children? One potential explanation is that helminths may differ in their susceptibility to IgE antibodies. More specifically, *T. trichiura *may be more resistant than *A. lumbricoides *to IgE-dependent effector mechanisms. Thus, the effect of *T. trichiura *on the immune system would enhance IgE responses in a whole, accounting for its association with atopic wheezing, without concomitantly leading to self-elimination.

## Conclusion

The present results show that helminthic infection may be, in addition to respiratory tract viruses, a cause of early wheezing in small children who have been recently or lightly infected. These helminthic infections may also be involved in increasing atopy. Further investigations, based on birth-cohort or experimental animal studies, should be performed in order to clarify the role played by these parasites, their soluble antigens, or anti- parasite - anti-allergen cross-reactive antibodies in the pathogenesis of atopy, asthma and other allergic disorders.

## List of abbreviation

CI: confidence interval; KU/L: kilo unit per liter; ASIGE: allergen specific immunoglobulin E antibody; ISAAC: international Study of Asthma and allergies in Childhood; OR: odds ratio; TH2: T helper cells 2; SCAALA: (Social Change, Asthma and Allergy in Latin America) research program;

## Conflict of interest

All authors report no conflict of interest financial or otherwise, with the findings of this study.

## Authors' contributions

NA-N has designed and wrote the manuscript; SB helped in the surveys and carried out the laboratory assays; MS has done the statistical analyses; LP-C helped in writing and reviewed the manuscript. MB has coordinated the group, conceived and reviewed the manuscript. All authors read and approved the final manuscript.
